# A Cross-Sectional Survey on Dietary Supplements Consumption among Italian Teen-Agers

**DOI:** 10.1371/journal.pone.0100508

**Published:** 2014-07-02

**Authors:** Valeria del Balzo, Valeria Vitiello, Alessia Germani, Lorenzo M. Donini, Eleonora Poggiogalle, Alessandro Pinto

**Affiliations:** Department of Experimental Medicine, Research Unit of Food Science and Human Nutrition, “Sapienza” University of Rome, Rome, Italy; Geisel School of Medicine at Dartmouth College, United States of America

## Abstract

**Introduction:**

In the last decades, dietary supplements consumption has increased in the Western world for all age groups. The long-term potentially dangerous effects related to an indiscriminate consumption of dietary supplements are still unknown and are becoming a matter of public health concern. Therefore, the aims of the present study were: to explore the contribution of dietary supplements to micronutrient daily intake, and to evaluate awareness and knowledge about dietary supplements.

**Methods:**

Participants (age ranging from 14 to 18 years) were recruited among students attending 8 high schools in the province of Frosinone (Italy). An anonymous questionnaire, composed of 12 multiple-choice items, was administered to all the participants. T-test and the analysis of variance (ANOVA) were performed to describe differences between means of the groups, while the chi-square test was used to compare observed and expected frequencies. The logistic regression model, aimed at identifying the characteristics of potential consumers of dietary supplements,

**Results:**

A total of 686 teenagers (288 males and 398 females, average age: 17,89±0,91 years) participated in the study. The 83,6% of participants affirmed to be aware of dietary supplements. 239 participants consumed dietary supplements: 118 males and 121 females. 49,1% of females consumed dietary supplements less than twice a week, whereas 43,6% of males consumed dietary supplements from 2 to 5 times per week. Statistically significant differences emerged between the genders with respect to the source of information regarding dietary supplements, the perceived indications for their use, and the choice of the store/place to purchase them.

**Discussion:**

Findings in the present study provide insight into the consumption of dietary supplements among young Italians, highlighting the need to foster further awareness among adolescents about the correct use of dietary supplements, especially in terms of indications and contraindications.

## Introduction

In the last decades, dietary supplements consumption has increased in the Western world in all the age groups, as a strategy for disease prevention, for the correction of inadequate lifestyle habits, and for the improvement of physical performance [1]–[3].

Dietary supplements are defined as concentrated sources of nutrients or other substances with a nutritional or physiological effect that increase the overall dietary intake by supplementing the normal diet. They are marketed in measured doses (i.e. as pills, tablets, capsules, liquids etc.) [4], [5].

Recent data provided by Bailey et al., estimating dietary supplement intake in the U.S. population using the NHANES 2003–2006, showed that any dietary supplement use was 26% in 14–18 year-old adolescents and was higher (43%) among 4–8 year-old children [2].

The use of dietary supplements is influenced by a number of factors such as gender, age, educational level, socio-economic status, place of residence, and ethnicity. A Korean survey involving healthy teenagers reported that supplement use was highest in high-school students, females, individuals living in rural communities, and in individuals with a high socio-economic status [3].

In the 2007 National Health Interview Survey, it emerged that dietary supplement use in US children, was more frequent in Asian, white, or non-Hispanic subjects, belonging to families with higher parental education and income levels, and being healthier than non-users [1].

The long-term and/or cumulative, potentially dangerous effects related to an indiscriminate consumption of dietary supplements containing micronutrients are still unknown [6], [7], and are becoming a public health concern. For this reason, in a number of countries, surveys were performed in order to investigate the consumption of dietary supplements in different age groups, the factors influencing their consumption and the profile of the typical consumer [8]–[10].

Nowadays, it is of concern that the “tolerable upper intake level” (UL) [11], for many nutrients could be overcome as consequence of the increased consumption of dietary supplements. The UL corresponds to the highest intake level which does not cause adverse effects on the health status in all the individuals belonging to a specific population group.

No current data exist for the Italian population with respect to dietary supplement consumption. We carried out a survey involving Italian teenagers in order to investigate the use of dietary supplements, and to evaluate the awareness and knowledge about dietary supplements in this age group.

## Materials and Methods

Participants (age ranging from 16 to 19 years old) were recruited from individuals attending 8 high schools in the province of Frosinone (Italy) and referring to health care services at the “Local Health Authority ASL Frosinone- Department B”.

The study was approved by the local ethics committee (Azienda policlinico Umberto I of Rome). The informed written consent was obtained by all the participants, or by their legal representatives. The participation was on voluntary basis.

A questionnaire was developed on the basis of existing questionnaires used in previous studies for the collection of information about dietary supplement consumption and lifestyle [13], [14]. The questionnaire was self-administered to all the participants, who answered it anonymously.

The questionnaire was composed of 12 multiple-choice items exploring the following domains:

- demographic characteristics;
- knowledge and information about dietary supplements (nutrition awareness);
- recall of dietary supplement use (frequency and quantity);
- reasons for dietary supplement use;
- spontaneous or prescribed/advised dietary supplement use (knowledge transfer);
- places to purchase dietary supplements;
- perceived effects on health status;
- future dietary supplement consumption;

For the purpose of this study, dietary supplements were divided into 7 categories: energizers (products with taurine, ornithine, tryptophan and B-group vitamins), amino acids and/or protein, vitamins, minerals, vitamins + minerals, fiber, sport drink (products with K, Mg, Cl, Na and maltodextrin) according to the classification used for pharmaceutics [15]. Also the trading name was asked for, in order to identify the kind of dietary supplement used.

### Statistical analysis

Collected data were analysed verifying the gender-related differences concerning awareness and knowledge about dietary supplements. In particular a logistic regression model was built to identify possible factors leading to a higher risk for dietary supplement consumption. For this purpose participants were divided into two age classes (16–17 and ≥18 years).

After verification of the normal distribution of the variables, t-test and the analysis of variance (ANOVA) were performed to describe differences between means of the groups, while the chi-square test was used to compare observed and expected frequencies.

Differences were considered to be statistically significant at p<0.05. Statistical analysis was performed using SPSS 10.0 statistical software (SPSS Inc. Wacker Drive, Chicago, IL, USA).

The logistic regression model, aimed at identifying the characteristics of potential consumers of dietary supplements, was obtained considering as primary dependent variable in outcome the use of dietary supplements, while controlling as co-variants the social and demographic variables which were singularly correlated with the primary response variable.

## Results

A total of 686 teenagers (288 males and 398 females, average age: 17.89±0.91 years) participated in the study. No significant differences were observed between the genders for age, school year, and types of high schools (table 1).

**Table 1:** Study population distribution by age and type of high school.

	Total participants	Males	Females
	(n. 686)	(n. 288)	(n. 398)
Age (years; mean ± SD)	17.89±0.91	17.93±0,92	17.86±0.90
Classic or Scientific High School (%)	71	69.8	71.9
Other types of High School (%)	29	30.2	28.1

According to Table 2, 83.6% of participants affirmed to be aware of dietary supplements. Statistically significant differences were observed between males and females in the awareness of the perceived indication of dietary supplement for their use, except for students providing the answer: “inadequate food intake”, or “equivalent of meal substitutes”, or “potentially dangerous if improperly used”, as perceived indication of dietary supplements for their use.

**Table 2:** Knowledge of dietary supplements and awareness of perceived indications for their use.

		Totalparticipants(n. 686)	Males(n. 288)	Females(n. 398)
Do you know dietarysupplements? (%)	Yes	83.6	83.4	83.5
	No	16.4	16.6	16.5
Perceived indicationof dietary supplementsfor their use	Nutritional deficiencies	36.8	31.3	40.7*
	Exercise training	37.4	45.2	31.6*
	Increased nutritionalrequirements	16.9	23.1	11.6*
	Inadequate food intake	37.4	33.7	40.4
	Prescription drugs	9.1	13.9	5.1*
	Equivalent ofmeal substitutes	6.6	4.3	8.4
	Potentially dangerous ifimproperly used	24.1	28.8	20.0

*p<0,05 statistically significant difference between males and females.

N.B.: Multiple choice was allowed.

**Figure 1:**
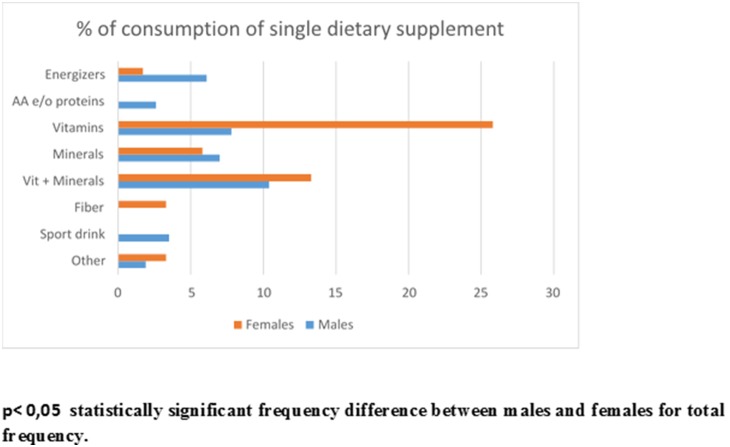
Distribution of the study population on the basis of the consumption of single dietary supplement type by category.

Table 3 summarizes the distribution of the study population on the basis of the dietary supplement use, and of the number of types of dietary supplement consumed. 239 participantsconsumed dietary supplements: 118 males (49%) and 121 females (51%). Most of the participants interviewed (38.3% of males and 53.3% females) consumed only one type of dietary supplement but a small proportion of participants consumed at least three different types of supplements. The distribution of the 239 participants,based on the consumption of single supplement type by category, is shown in Figure 1.

**Table 3:** Distribution of the study population on the basis of the number of types of consumed dietary supplements.

	Total users (n = 239) (%)	Males (n = 118) (%)	Females (n = 121) (%)
1	46.0	38.3	53.3*
2	31.7	31.3	32.5
3	16.0	20.9	10.8*
>3	6.3	9.5	3.4*

*p<0,05 statistically significant difference between males and females.

Statistically significant differences (p<0.05) emerged between the genders with respect to the knowledge transfer and suggestions for use about dietary supplements and place to purchase them. Males used dietary supplements under the advice of friends (14.9%), physician (14.8%), physical trainer (16.7%), chemist (5.3%), or other non-specified figures (43.0%). They bought dietary supplement at the drugstore (39.8%), at the supermarket (23.9%), at the gym (13.3%), or other stores (23%). On the other hand, females used dietary supplements under the suggestion of family (19%), physician (43.1%), friends (5.2%), physical trainer (6.9%), chemist (5.2%), or other non- specified subjects (20.6%). They purchased dietary supplement mainly at the drugstore (69.0%), at the supermarket (14.7%), at the gym (5.2%) or other stores (11.1%).

In Table 4, the frequency of consumption of dietary supplements is shown. Most females (49.1%) consumed dietary supplements less than twice a week, whereas the majority of males (43.6%) consumed dietary supplements from 2 to 5 times per week, this latter data is statistically significant (p<0.05).

**Table 4:** Frequency of consumption.

	Total users (n = 239) (%)	Males (n = 118) (%)	Females (n = 121) (%)
More than 5 times a week	13.7	10.9	16.7
2–5 times a week	27.9	43.6	12.3*
Less than twice a week	43.4	37.3	49.1
Periodically during the year	11.9	6.4	17.5*
More than one of the previous answers	3.1	1.8	4.4*

*p<0,05 statistically significant frequency difference between males and females.

In Table 5, reasons for consumption are reported. The statistically significant (p<0.05) reasons for dietary supplements consumption were: the reintegration of mineral loss through sweat after physical training for males and the toning of the body (45.5%) and the health improvement (41.6%) for females.

**Table 5:** Reasons for consumption.

	Total users (n = 239) (%)	Males (n = 118) (%)	Females (n = 121) (%)
Toning the body	36.0	29.2	45.5*
Improving muscle mass	12.7	21.1	4.4*
Reintegrating mineral loss through sweat	36.0	52.2	20.4*
Improving Sport performance	21.9	33.6	10.6*
Improving Brain performance	7.0	5.3	8.8
Losing Weight	5.7	1.8	9.7*
Keeping Weight	4.8	4.4	5.3
Aesthetic reasons	6.1	6.1	5.3
Health reasons	28.9	16.8	41.6*

*p<0,05 statistically significant frequency difference between males and females.

**Multiple choice is allowed.

80.7% of males and 88.3% of females reported beneficial effects due to the consumption of dietary supplements, and 73.6% of males and 84.7% of females affirmed to be in favor of future consumption of dietary supplements, when necessary (Table 6) (p≤0.05).

**Table 6:** Benefic effects and willingness to continue assumption.

		Total users (n = 239) (%)	Males (n = 118) (%)	Females (n = 121) (%)
Have you been beneficied? (%)	YES	84.6	80.7	88.3
Would you continue to assume (%)	Always	9.4	13.6	5.4*
	Periodically	11.2	12.7	9.9*
	If necessary	79.4	73.6	84.7*

*p<0,05 statistically significant frequency difference between males and females.

## Discussion

In the current study we explored dietary supplement consumption among Italian adolescents attending high schools in the province of Frosinone (central Italy).

We found that 35% of the interviewed teenagers consumed dietary supplements; our findings are similar to data provided by a Korean study reporting that 31% of students attending high schools used dietary supplements [3], as well as data from the NHANES 1999–2000, prevalence of dietary supplement use was 27.4% and 32.4% in 16–19 year-old males and females, respectively. [16] Results in our study, with respect to reasons for consumption and knowledge transfer about dietary supplements, are consistent with those reported in previous studies, observing that dietary supplementation was found to be popular overall among adolescents engaged in exercise training, searching for the improvement of physical performance and the increase of muscle mass. In addition, the source of information regarding dietary supplements was analogous in existing studies, represented mainly by coaches and friends [17], [18] This observation is worrisome because dietary supplementation should be started under the prescription and the supervision of health care professionals (e.g. nutritionist, dietitian, general practitioner, etc.), after a thorough evaluation of clinical and nutritional status, including biochemical tests assaying vitamin and mineral levels, when suspecting a specific deficiency.

The majority of teenagers involved in our study reported beneficial effects from dietary supplement intake. This finding highlights the need to foster further awareness among adolescents about the correct use of dietary supplements, especially in terms of indications and contraindications; the perceived benefits could underestimate potential risks and side effects related to the inappropriate consumption as well as to overconsumption of dietary supplements. In particular, a wealth of studies described negative and dangerous effects of nutritional supplementation in children and adolescents. [18]–[20]. Moreover, in different countries, intake of vitamins and minerals was frequently found to exceed the tolerable upper intake level in many age groups in children and adolescents [2], [21]–[24].

Potential toxicity of large doses of vitamins may encompass not only accumulation problems, but also the exaggeration of oxidative stress. The overcoming of tolerable upper intake level for the fat-soluble vitamins may lead to toxicity phenomena, due to accumulated micronutrients within adipose tissues. Furthermore, high doses of vitamin E exert a pro-oxidant effect and influence apoptosis [25].

Toxicity concerns were reported also for hydro-soluble vitamins; *in vitro* studies showed that high levels of vitamin C in presence of transition metals (i.e. iron) were able to convert ascorbic acid from an anti-oxidant to a pro-oxidant agent, promoting the reactive oxygen species (ROS) generation. [25].

From the logistic regression analysis, aimed at identifying the characteristics of potential consumers of dietary supplements, it emerged that the typical profile of an adolescent consumer corresponded to a male attending technical high schools and consuming more than one dietary supplement simultaneously, mainly during exercise training with the aim of increasing muscle mass, under the advice of the physical trainer or friends. Completely different was the profile of young female consumers, represented by teenagers attending scientific or classical high schools, consuming one type of dietary supplements for health purposes or aesthetic reasons, and buying them in the drug-store under the advice of a physician or family.

As emerged in the present study, and in agreement with the extant literature, the profile of the consumer of dietary supplements corresponded to a teenager in a good health status, regularly engaged in exercise training, consuming good quality foods and belonging to the medium- upper class [21], . These characteristics seem to be in contrast with indications of nutritional supplementation, mainly addressed to the compensation of micronutrient deficiencies due to an inadequate diet, lack of appetite, or concomitant diseases [28].

Some limitations of the current study need to be taken into account. This study was carried out in just few high schools in a limited area in the centre of Italy, hence results are not fully generalizable. In addition, we did not evaluate exact doses consumed for the different dietary supplements nor duration of use by participants. No information was available regarding parameters or biomarkers of nutritional status. Finally, the use of multiple-choice items could have prevented the collection of other information about nutritional supplementation in our study population.

As the consumption of dietary supplements among teenagers is an increasing phenomenon in the developed countries, it is important to clarify for which nutrients the tolerable upper level could be most frequently exceeded in case of dietary supplement consumption, in order to reduce potential toxicity. Further research should be prompted, encompassing a larger variety of dietary supplements, with new nutrients profiles, involving a larger study population, exploring nutritional supplementations as well as food habits, health and nutritional status.

Although, to date, a universal consensus does not exist about benefits or adverse effects related to the use of dietary supplements in healthy individuals, it should be remarked in the young as well as in the adult population (especially among parents and health educators) that, under physiological conditions, an adequate, balanced and varied diet, combined with an appropriate nutritional education, is sufficient to provide all the nutrients necessary to the individual’s correct development and to allow the maintenance of a good health status [29], [30].
